# Redox Network Dysfunction: Integrating Ferroptosis and Cuproptosis Across Human Diseases

**DOI:** 10.3390/antiox15010024

**Published:** 2025-12-23

**Authors:** Federica Li Pomi, Guglielmo Di Leo, Sara Genovese, Francesco Borgia, Sebastiano Gangemi

**Affiliations:** 1 Department of Precision Medicine in Medical, Surgical and Critical Care (Me.Pre.C.C.), University of Palermo, 90127 Palermo, Italy; 2 Institute of Polar Sciences (IPS), National Research Council of Italy (CNR), 98164 Messina, Italy; guglielmo.dileo@cnr.it; 3 Institute for Biomedical Research and Innovation (IRIB), National Research Council of Italy (CNR), 98164 Messina, Italy; sara.genovese@cnr.it; 4 Section of Dermatology, Department of Clinical and Experimental Medicine, University of Messina, 98125 Messina, Italy; fborgia@unime.it; 5 Allergy and Clinical Immunology Unit, Department of Clinical and Experimental Medicine, University of Messina, 98125 Messina, Italy; sebastiano.gangemi@unime.it

**Keywords:** ferroptosis, cuproptosis, oxidative stress, lipid peroxidation, mitochondrial stress, reactive oxygen species, malondialdehyde, lipidomics, inflammation, artificial intelligence

## Abstract

Oxidative stress (OS) is increasingly recognized as a dynamic disturbance of cellular redox networks rather than a simple imbalance between oxidants and antioxidants. In this context, ferroptosis and cuproptosis—two regulated and metal-dependent forms of cell death—emerge as key mechanisms linking OS to metabolic dysfunction, inflammation, and tissue injury. This review integrates findings from biochemical, lipidomic and metallomic studies to describe how lipid peroxidation (LPO), glutathione (GSH)–Glutathione Peroxidase 4 (GPX4) activity, ferritinophagy, copper-induced mitochondrial protein lipoylation, and altered communication between organelles generate distinct redox signatures across diseases. By examining cutaneous, metabolic, cardiovascular, infectious, neurodegenerative, and oncologic conditions, we outline the shared redox pathways that connect iron- and copper-dependent cell death to systemic inflammation, immune dysregulation, and chronic tissue damage. Common oxidative markers—such as oxidized phospholipids, lipid aldehydes including 4-Hydroxynonenal (4-HNE) and malondialdehyde (MDA), and systemic metal imbalance—are highlighted as potential indicators of disease severity and as emerging therapeutic targets. We also discuss innovative analytical tools, including redox lipidomics, metallomic profiling and artificial-intelligence (AI)-based classification approaches, which improve the characterization of redox vulnerability and may guide the development of precision redox therapies. Overall, ferroptosis and cuproptosis represent unifying mechanisms that connect OS to multisystem disease and provide new opportunities for diagnostic refinement and targeted antioxidant-based interventions.

## 1. Introduction

Over the past decades, the concept of OS has undergone a crucial transformation. Originally defined as an imbalance between the production of reactive oxygen and nitrogen species (ROS and RNS) and the antioxidant defence system, OS was long regarded as a purely destructive process leading to LPO, protein oxidation, and DNA damage [1,2]. However, advances in redox biology have revealed a more complex reality. Rather than a unidirectional cause of damage, redox reactions seem to form part of a dynamic communication network that orchestrates metabolism, immunity, and cell biology across different cellular compartments and tissues [1].

This new perspective suggests that controlled ROS and RNS pulses are indispensable for physiological signalling [3]. When spatially and temporally restricted, they modulate kinases, transcription factors, and epigenetic enzymes to regulate processes such as cell proliferation, differentiation, and immune activation. When this compartmental control is lost, oxidants become pathological, converting physiological eustress into oxidative distress with systemic consequences. Such transitions may lead to a broad spectrum of human disorders—from skin barrier dysfunction and endocrine autoimmunity to metabolic inflammation and cancer development [4,5]. At the cellular level, mitochondria, peroxisomes, and the endoplasmic reticulum maintain distinct redox microenvironments that communicate through diffusible oxidants. The integrity of this organellar dialogue is essential for metabolic homeostasis. When disrupted, oxidative injury propagates between compartments, reprogramming metabolism and immune responses in ways that cannot be explained solely by oxidant excess [6]. In this context, OS may no longer be viewed merely as biochemical damage but rather as a disturbance of a highly coordinated redox network.

Within this framework, the “redox network axis” can be viewed as an integrated system comprising redox-active organelles—such as mitochondria, peroxisomes, lysosomes, the endoplasmic reticulum, and the plasma membrane—together with transition-metal pools (labile Fe^2+^/Fe^3+^ and Cu^+^/Cu^2+^), low–molecular-weight redox couples, and oxidizable lipid substrates, particularly polyunsaturated fatty acids (PUFAs)-containing phospholipids. These components collectively maintain local redox balance but also provide the biochemical substrates for oxidative reactions [7,8,9]. Redox network dysfunction may emerge when coordination among these elements breaks down—through the expansion of labile metal pools, reduced antioxidant buffering, or aberrant accumulation and remodelling of PUFA-rich phospholipids—thereby creating conditions that favour metal-dependent regulated cell death [10].

Clinical and translational studies across dermatology, endocrinology, autoimmunity, and haematology have been crucial in driving this conceptual transition. Early investigations used classical oxidative markers—including malondialdehyde (MDA), advanced oxidation protein products (AOPPs), and advanced glycation end products (AGEs)—to quantify damage [11,12]. Later, it became evident that these molecules actively modulate cellular pathways: oxidative biomarkers (MDA, AOPPs, and AGEs) correlate with cytokine release, immune-cell activation, and mitochondrial stress, suggesting that these species act as functional mediators rather than passive byproducts of oxidative damage [3].

An additional layer of complexity in redox biology arises from the exposome, namely the cumulative set of environmental, lifestyle-related, and endogenous exposures that shape human physiology across the lifespan [13,14]. Increasing evidence has highlighted that many external exposures—air pollutants, heavy metals, pesticides, microplastics, and industrial solvents—are potent drivers of oxidative imbalance, enhancing ROS production, disrupting mitochondrial respiration, and accelerating LPO onset, thereby promoting chronic inflammation, cellular senescence, and tissue vulnerability [15]. In parallel, internal exposome components such as diet, microbiome-derived metabolites, and chronic inflammatory tone interact with these environmental inputs to modulate redox status at both systemic and organ-specific levels [16,17,18].

Environmental toxicants also intersect with genetic susceptibility, creating a redox-sensitive interface that influences the risk of neurodegenerative and metabolic disease. Persistent exposure to pesticides, metals, and organic pollutants has been linked to increased incidence of Parkinson’s disease, Alzheimer’s disease, and amyotrophic lateral sclerosis, in part through impaired detoxification pathways, mitochondrial dysfunction, and destabilization of antioxidant networks [15,19].

At the organismal scale, chronic exposome-induced oxidative pressure progressively erodes redox resilience by suppressing nuclear factor erythroid 2-related factor 2 (Nrf2)-dependent cytoprotective programs, altering DNA and histone methylation, impairing stem cell function, and amplifying low-grade systemic inflammation [20]. Such processes accelerate biological ageing trajectories and heighten susceptibility to ferroptosis, cuproptosis, and other metal-dependent forms of regulated cell death [15].

Moving to skin disorders, such as psoriasis and atopic dermatitis (AD), OS has been demonstrated to contribute to inflammation and epidermal hyperproliferation by activating Nuclear Factor kappa-light-chain-enhancer of activated B cells (NF-κB) and Signal Transducer and Activator of Transcription 3 (STAT3), disrupting the skin barrier, and finally amplifying cytokine networks [21,22,23,24]. In autoimmune thyroid diseases, oxidative and glycoxidative alterations of thyroglobulin appear to increase its antigenic potential, thus sustaining autoreactive lymphocyte activation and perpetuating autoimmune inflammation [25,26,27]. Likewise, in haematologic malignancies, cancer cells exploit moderate ROS levels for growth signalling while upregulating antioxidant defences to avoid lethal oxidative injury, thereby transforming redox balance into a survival advantage and potential therapeutic vulnerability [28,29].

Parallel mechanistic advances have extended the redox framework beyond classical OS to include regulated cell-death pathways. Two paradigms—ferroptosis and cuproptosis—illustrate how metal homeostasis, LPO, and mitochondrial dysfunction are integrated within the redox network [30]. Ferroptosis, an iron-dependent form of regulated cell death, occurs when cystine (Cys) import or GSH synthesis is impaired or when glutathione peroxidase 4 (GPX4) is inhibited. These events allow the accumulation of lipid hydroperoxides, which compromise membrane integrity in the cells [31,32]. In contrast, cuproptosis is triggered by copper overload, which targets lipoylated tricarboxylic-acid enzymes, induces proteotoxic aggregation, and disrupts mitochondrial respiration [33]. Both processes highlight how essential transition metals can switch from signalling cofactors to lethal catalysts when redox regulation fails.

Redox balance also exerts a deep influence on the immune system. Reactive species shape immune responses through redox-sensitive transcriptional switches, thus influencing antigen presentation and T-cell differentiation [34,35]. Oxidized phospholipids and aldehydes produced during ferroptosis can act as endogenous adjuvants, enhancing dendritic-cell maturation and antitumour immunity, while excessive oxidation can impair peptide–MHC stability and T-cell priming [36,37]. These observations may highlight that redox tone must be modulated with contextual precision rather than suppressed indiscriminately by antioxidant therapy.

**Figure 1:**
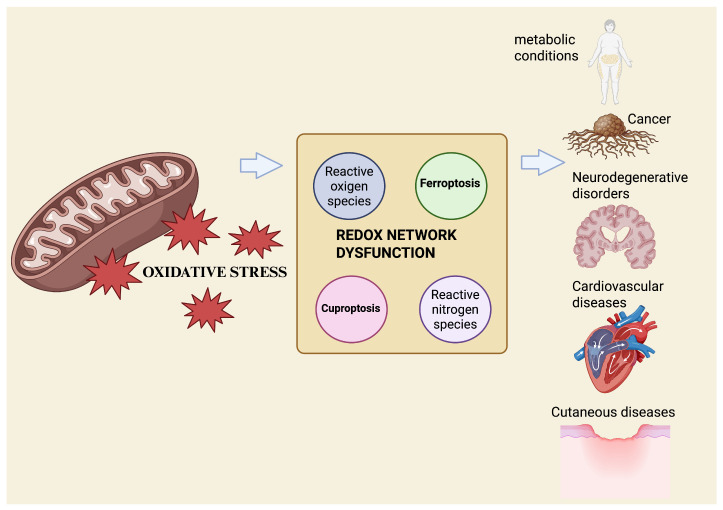
Mitochondrial OS acts as an upstream driver of redox-network dysfunction, promoting metal-dependent regulated cell death. Excess ROS disrupt iron and copper homeostasis, deplete antioxidant defenses, and favor ferroptosis and cuproptosis, contributing to multisystem disease pathogenesis. Created with BioRender.com.

Recent approaches, such as redox lipidomics, which enables large-scale profiling of oxidized lipid species, and metallomics, the system-wide analysis of biologically relevant metal species, allow the simultaneous quantification of oxidized lipids, metal ratios, and key redox couples, including GSH/glutathione disulfide (GSSG) and nicotinamide adenine dinucleotide phosphate in its reduced and oxidized forms (NADPH/NADP^+^). Together, these technologies support the emergence of redox phenotyping as a translational tool [38,39]. The integration of these datasets with machine-learning models trained on ferroptosis and cuproptosis gene signatures has begun to classify tissues and diseases based on their redox vulnerability and predict therapeutic responses in real-time [40] (Figure 1).

This review summarizes emerging evidence supporting the concept of “redox network dysfunction” as a unifying mechanism linking OS, metal-dependent cell death, and immunometabolic reprogramming across human diseases. By integrating mechanistic and clinical insights, this review aims to provide a coherent framework that connects redox biology with inflammation, immunity, and future therapeutic innovation.

## 2. Molecular Mechanisms of Cell Death Pathways: Ferroptosis and Cuproptosis

Ferroptosis is a regulated form of cell death characterized by iron-dependent LPO that overwhelms antioxidant defences. Unlike apoptosis or necroptosis, it is driven by metabolic imbalance rather than receptor or caspase activation [41]. The process begins when intracellular GSH becomes depleted or when glutathione peroxidase 4 (GPX4) activity is insufficient to detoxify phospholipid hydroperoxides [42]. As shown in recent studies, lipidomic analyses reveal that polyunsaturated phospholipids are the key targets of oxidation, linking altered lipid metabolism to the onset of ferroptosis [43]. In this context, the enzyme acyl-CoA synthetase long-chain family member 4 (ACSL4) has been demonstrated to promote the incorporation of long-chain PUFAs into membrane phospholipids, creating substrates that are highly sensitive to peroxidation [44]. Mitochondria and peroxisomes contribute to this process by generating oxidized intermediates and ROS that amplify LPO and oxidative injury [45,46]. During ferroptosis, mitochondria display characteristic structural alterations, including progressive shrinkage, increased membrane density, and loss of cristae architecture. These features represent the distinctive morphological hallmarks of this form of cell death [47].

The sensitivity of a cell to ferroptosis depends on the balance between oxidant generation and antioxidant systems. The canonical GPX4–GSH axis is the first line of defense, reducing lipid hydroperoxides to alcohols, which are innocuous compounds. When the import of Cys through the system Xc^−^ transporter is blocked or when GSH synthesis is diminished, this protection collapses, and lipid peroxides begin to accumulate in the cells. System Xc^−^ is a heterodimeric Cys/glutamate (Glu) antiporter composed of the light chain subunit SLC7A11 (xCT), which mediates substrate specificity and transport activity, and the heavy chain subunit SLC3A2 (4F2hc), which is required for membrane localization and transporter stability.

In parallel with this GPX4-dependent layer, cells also rely on GSH -independent mechanisms, such as the ferroptosis suppressor protein 1 (FSP1)-coenzyme Q10 (CoQ10)-NADPH axis at the plasma membrane, which regenerates reduced CoQ10 to neutralize lipid radicals, and mitochondrial dihydroorotate dehydrogenase (DHODH), which maintains redox balance by producing ubiquinol to prevent LPO [48,49]. These pathways constitute compartmentalized antioxidant layers that collectively restrain phospholipid peroxidation, and their contribution to ferroptosis resistance is not contingent upon GPX4 inactivation [50]. Together, these systems expand the cell’s antioxidant capacity and transiently delay ferroptosis [51].

Once these defences fail, LPO becomes self-sustaining, defining the “point of no return” beyond which cell viability cannot be restored [43]. Iron metabolism is a key determinant of when and where ferroptosis occurs. Within cells, most iron is securely stored in ferritin, while a smaller labile iron pool engages in redox-active reactions. When ferritin undergoes selective autophagic degradation (ferritinophagy), ferrous iron (Fe^2+^) is released, enlarging the labile pool and driving Fenton-type radical formation that triggers LPO and thereby promotes ferroptosis [52,53]. Beyond ferritinophagy, other pathways expand the intracellular Fe^2+^ pool. Transferrin receptor-mediated endocytosis increases endosomal Fe^3+^, which is reduced to Fe^2+^ by Six-Transmembrane Epithelial Antigen of Prostate 3 (STEAP3) and subsequently transferred to the cytosolic labile iron pool. Mitochondrial iron-sulfur (Fe-S) clusters, being highly susceptible to oxidative damage, become destabilized under stress conditions, releasing labile iron into the matrix and thereby amplifying ROS production and LPO [54]. Stress-induced activation of heme oxygenase-1 (HO-1), encoded by the HMOX1 gene, liberates Fe^2+^ from heme-containing proteins, whereas lysosomal degradation of iron-binding proteins further contributes to fluctuations in redox-active iron. HO-1 is a central stress-responsive regulator of ferroptosis, exerting context-dependent protective or pro-ferroptotic effects. By catalyzing heme degradation, HO-1 generates biliverdin/bilirubin and carbon monoxide, which display antioxidant and cytoprotective properties, but simultaneously, it releases Fe^2+^, thereby expanding the labile iron pool. Under conditions of limited antioxidant capacity or excessive OS, HO-1-derived Fe^2+^ can fuel Fenton chemistry, promote LPO, and lower the threshold for ferroptotic cell death. Experimental studies demonstrate that HO-1 induction enhances ferroptosis in response to system Xc^−^ inhibition or GPX4 inactivation, whereas genetic or pharmacological inhibition of HO-1 attenuates ferroptotic injury in several models. Conversely, when ferritin induction and redox buffering prevail, HO-1 may limit ferroptosis by facilitating iron sequestration and reducing free heme–mediated oxidative damage [55,56,57]. Collectively, these processes establish a ferroptosis-permissive redox landscape in which phospholipid peroxidation can exceed the capacity of antioxidant defence systems [41,53].

Mitochondria are central to this process, acting as both a source and a target of OS. Electron leakage from the respiratory chain and the instability of Fe-S clusters enhance ROS generation, further damaging mitochondrial membranes and enzymes [58]. Beyond mitochondria, other organelles also shape ferroptotic sensitivity: lysosomes regulate intracellular iron availability, peroxisomes influence the oxidation of PUFAs, and ER–mitochondria contact sites coordinate lipid metabolism and redox signalling. Together, these organelle interactions appear to influence cell- and tissue-specific vulnerability thresholds, suggesting that ferroptosis may arise not simply through a linear sequence of events but through the disruption of interconnected redox processes across multiple cellular compartments [59,60,61].

**Figure 2:**
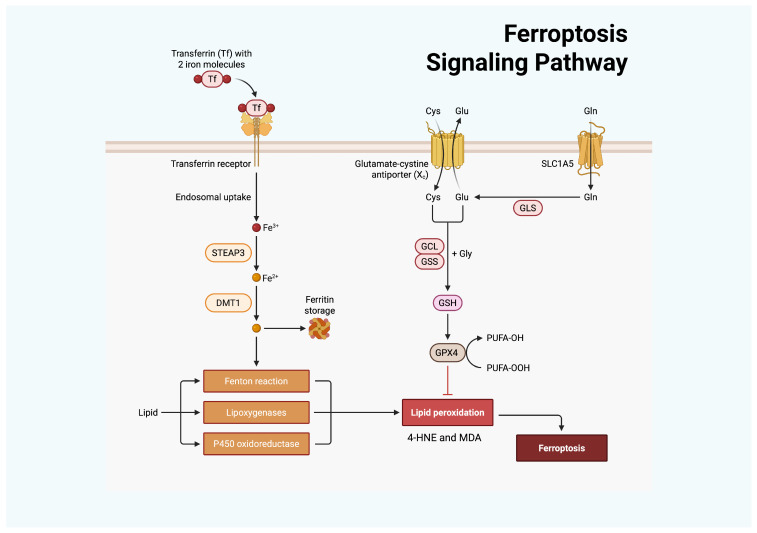
Ferroptosis is regulated by cellular iron and antioxidant metabolism. Transferrin delivers Fe^3+^ via the transferrin receptor, after which STEAP3 reduces it to Fe^2+^, and divalent metal transporter 1 (DMT1) mediates cellular import. Accumulated Fe^2+^ promotes OS through the Fenton reaction, lipoxygenases, and P450 oxidoreductase, thereby enhancing LPO. In parallel, Cys uptake through the Xc^−^ antiporter supports GSH synthesis via GCL and glutathione synthetase (GSS). GSH serves as a substrate for GPX4 to detoxify lipid peroxides, with glutaminase (GLS)–derived Glu contributing to this process. GPX4 inhibition or insufficiency results in uncontrolled LPO and ferroptotic cell death. Created with BioRender.com.

Beyond metabolism, ferroptosis is increasingly recognized as a modulator of immune function across infections, autoimmunity, and cancer. In infectious settings, ferroptosis may either restrict pathogens or be co-opted by them, although current evidence remains heterogeneous and mechanistically incomplete. In autoimmune disease, enhanced LPO and reduced antioxidant defenses suggest a contribution of ferroptosis to tissue injury, but its relative weight compared with other death pathways is still unresolved. In cancer, T-cell-driven ferroptosis in tumor cells can enhance immunity, yet ferroptosis in immune cells may impair responses, underscoring the intrinsically dual role of ferroptosis in tumor–immune dynamics and the need to clarify its context-dependent effects [62,63,64] (Figure 2).

Cuproptosis, recently described by Tsvetkov, represents a parallel form of metal-dependent cell death, centred on copper toxicity and mitochondrial dysfunction [65]. Copper is essential for mitochondrial energy metabolism but becomes cytotoxic when it accumulates beyond physiological limits. This occurs as excess copper binds to lipoylated enzymes of the tricarboxylic acid (TCA) cycle—particularly dihydrolipoamide S-acetyltransferase (DLAT)—causing abnormal protein aggregation, loss of Fe-S clusters, and collapse of mitochondrial respiration [65]. This mechanism depends on ferredoxin 1 (FDX1), which controls copper reduction and the lipoylation of mitochondrial proteins. When this pathway is perturbed, copper accumulates in the mitochondria, leading to proteotoxic stress, impaired oxidative phosphorylation, and loss of bioenergetic control [51]. The fine balance between copper import, utilization, and detoxification thus defines a narrow window between essential redox signalling and toxicity. In physiological settings, tight control of copper trafficking—coordinated by transporters such as CTR1, ATP7A, and ATP7B—maintains this balance and protects cells from cuproptotic stress. Disruption of these pathways seems to contribute to several human diseases. For example, in Wilson’s disease, defective ATP7B-mediated copper export leads to toxic hepatic and neurological accumulation. Beyond genetic disorders, altered copper handling is increasingly implicated in cancer, where elevated intracellular copper may influence tumour growth and metabolic vulnerability to cuproptosis. However, in vivo evidence remains limited, and the precise contribution of cuproptosis to disease progression requires further mechanistic clarification [66].

Although ferroptosis and cuproptosis are triggered by different metals and occur in distinct compartments—iron-driven lipid oxidation in membranes versus copper-driven protein aggregation in mitochondria—they are suggested to converge mechanistically on the metal–redox axis [67,68].

Both reveal how transition metals, indispensable for energy metabolism and enzyme catalysis, become cytotoxic when compartmental control fails. These pathways seem to interact functionally. Evidence suggests that copper and iron metabolism can influence each other’s redox activity, creating conditions that favor OS, although direct mechanistic convergence between cuproptosis and ferroptosis remains only partially defined [69].

Moreover, altered copper availability may disturb mitochondrial function and cellular redox balance, indirectly affecting pathways related to iron handling and LPO [70].

The immunometabolic nature of ferroptosis and cuproptosis extends their relevance across clinical contexts. Ferroptosis contributes to tissue injury in ischemia–reperfusion, neurodegeneration, and inflammatory dermatoses, but can also be therapeutically harnessed to eliminate apoptosis-resistant cancer cells [71,72,73]. Conversely, cuproptosis has emerged as a determinant of tumor progression and metabolic pathology in conditions of altered copper homeostasis [67,74,75]. In malignancies such as hepatocellular carcinoma and glioblastoma, preclinical data indicate that copper accumulation intersects with ferroptotic and cuproptotic pathways, shaping redox stress and, in some settings, sensitivity to antitumor immunity [66].

Although ferroptosis and cuproptosis are mechanistically distinct, multi-omic and experimental studies now support their convergence at the level of mitochondrial metabolism, GSH-dependent redox buffering, and metal-ion homeostasis. In several cancers, combined ferroptosis–cuproptosis signatures stratify tumor microenvironment (TME) phenotypes and therapeutic responses, suggesting that these death programs act both independently and through disease-specific crosstalk, particularly in malignancy [67,68]. These observations collectively support the hypothesis of a dual therapeutic paradigm: inducing ferroptosis or cuproptosis to suppress malignancy, or preventing excessive redox activation to protect against degenerative and inflammatory diseases.

Therapeutically, manipulating these metal-dependent processes offers new opportunities. Combined modulation of iron and copper fluxes could enable redox-guided precision therapy, in which cellular vulnerability is defined by the interplay among metals, lipids, and mitochondria [43]. Ultimately, ferroptosis and cuproptosis may exemplify the same principle: when communication between essential metals and organelles fails, oxidative signalling transforms from an adaptive mechanism into an irreversible death pathway.

## 3. Role in Human Diseases

### 3.1. Autoimmune and Endocrine–Metabolic Diseases

Redox imbalance represents a central pathogenic driver in diabetes mellitus, arising from sustained hyperglycemia that enhances mitochondrial ROS generation, activates Poly (ADP-ribose) Polymerase-1 (PARP-1), and inhibits glyceraldehyde-3-phosphate dehydrogenase, leading to upstream accumulation of glycolytic intermediates and stimulation of pro-oxidative pathways such as AGE formation [76]. These mechanisms foster persistent LPO, which amplifies damage to membranes, proteins, and DNA, contributing to β-cell dysfunction and endothelial injury [77]. In diabetes, chronic ROS elevation and impaired antioxidant defenses further intensify inflammatory signaling and promote carbonyl stress, which synergizes with LPO to accelerate microvascular and macrovascular complications typical of the diabetic condition [78]. Ferroptosis is increasingly recognized as a potential contributor to β-cell failure and diabetic tissue damage. Dysregulated iron metabolism, enhanced Fenton chemistry, and PUFA-rich phospholipid peroxidation driven by ACSL4, LPCAT3, and LOX enzymes sensitize cells in diabetes models to ferroptotic death, suggesting a link between iron overload, mitochondrial dysfunction, impaired insulin biosynthesis, and disease progression [79]. Moreover, ferroptosis seems to play a role in the pathogenesis of diabetic complications—including nephropathy, retinopathy, cardiomyopathy, and neuropathy—through ROS-dependent lipid oxidation and inflammatory amplification [80]. Cuproptosis also interfaces mechanistically with OS in diabetes. Copper accumulation promotes aberrant oligomerization of lipoylated mitochondrial proteins, loss of Fe-S clusters, depletion of GSH, and excessive ROS production, culminating in proteotoxic stress and mitochondrial failure. Dysregulation of copper-handling proteins such as ATOX1, ATP7A/B, FDX1, and SLC31A1 has been implicated in diabetic pathophysiology, indicating that cuproptosis-associated metabolic vulnerability may contribute to β-cell injury and broader metabolic derangements [81]. Together, the above findings suggest that ferroptosis and cuproptosis do not operate as isolated redox-injury programs in diabetes, but rather as parallel outputs of a shared metabolic–mitochondrial vulnerability. Hyperglycemia-driven ROS elevation, GSH depletion, altered iron handling, and mitochondrial dysfunction create a biochemical environment permissive to both iron-dependent LPO and copper-dependent proteotoxic stress [79,80,81,82,83,84,85]. Finally, the coexistence of iron overload, Fe-S cluster instability, and copper mismanagement suggests that β-cells and endothelial cells may experience overlapping ferroptotic and cuproptotic pressures, although direct mechanistic interactions remain incompletely defined.

Moving to thyroiditis, since thyroid hormone synthesis depends on oxidative iodination, the thyroid gland is prone to OS. Under physiological conditions, the generation of ROS required for hormone biosynthesis is balanced by a robust antioxidant defence system that includes superoxide dismutase (SOD), glutathione peroxidase (GPx), and catalase (CAT) [86]. In Hashimoto’s thyroiditis (HT), this equilibrium is disrupted. Several studies have documented enhanced LPO, protein oxidation, elevated nitrite and myeloperoxidase levels, along with reduced antioxidant enzyme [87,88]. The resulting oxidative burden damages lipids, proteins, carbohydrates, and nucleic acids, ultimately driving thyrocyte apoptosis. Among the main contributors, nicotinamide adenine dinucleotide phosphate (NADPH) oxidase plays a pivotal role in the excessive ROS generation observed in overactivated T and B lymphocytes [87]. Clinical investigations further support systemic OS in HT, reporting significantly elevated circulating levels of MDA compared with healthy controls [89,90]. The pathogenic cascade in HT arises from the interplay between oxidative injury and immune activation. Oxidative modifications of thyroglobulin (Tg) and thyroid peroxidase (TPO) seem to produce neoantigens that elicit autoimmune responses. Concurrently, ROS amplify inflammation by activating toll-like receptors (TLRs) and promoting Th1- and Th17-driven immune pathways [87]. Thyroid-stimulating hormone (TSH) itself exacerbates this cycle by stimulating additional ROS production, leading to further protein carbonylation and LPO, which perpetuate glandular damage.

### 3.2. Cutaneous Disorders

The skin is both a target and a driver of systemic redox imbalance. As a redox-active organ, it is constantly exposed to environmental stressors such as ultraviolet radiation (UVR), ozone, and pollutants, which induce ROS production [91]. Under physiological conditions, controlled oxidative flux regulates differentiation and barrier renewal, but persistent redox activation leads to inflammation and cell death [92]. Moving to inflammatory dermatoses, in AD, OS, and LPO have been demonstrated to play a role in the pathogenesis and exacerbation of the disease [93].

Animal studies highlight that OS disrupts both the epidermal and dermal microenvironments through multiple mechanisms. In epidermal keratinocytes, LPO induces direct injury to DNA, enzymes, and cellular membranes, while oxidation of proteins and lipids within the stratum corneum alters barrier integrity and worsens the manifestations of atopic eczema [23,94]. Recent multi-omics and mechanistic studies highlight that both ferroptosis and cuproptosis contribute to the immunometabolic dysfunction underlying AD. Ferroptosis-related alterations—including impaired GPX4 and SLC7A11 activity, NADPH oxidase-derived ROS, and dysregulated LPO pathways—compromise keratinocyte integrity, promote DAMP release, and potentiate type-2 inflammatory signalling [95]. Parallel genomic analyses reveal that cuproptosis-related genes and copper-handling programs also associate with immune-cell infiltration and AD molecular subtypes [96,97]. Mechanistic work in mouse models demonstrates that activation of a SLC31A1–α-ketoglutarate–KDM5B axis suppresses ferritin heavy chain 1 (FTH1), heightens keratinocyte susceptibility to oxidative cell death, and exacerbates AD-like inflammation, while genetic or pharmacologic blockade of this pathway attenuates skin lesions [98]. Together, these findings suggest that ferroptotic and cuproptotic vulnerabilities converge to amplify cutaneous inflammation in AD and may represent actionable metabolic targets in preclinical settings.

In psoriasis, ferroptosis is more directly characterized. Lesional skin displays enhanced lipid oxidation, increased 4-HNE, reduced GPX4, and altered expression of ACSL4, PTGS2, transferrin receptor, and ferritin subunits, indicating iron-dependent LPO and impaired antioxidant defence in keratinocytes [1,99,100]. Inhibition of keratinocyte ferroptosis, for example, with ferrostatin-1, has been demonstrated to alleviate psoriasiform inflammation in experimental models, although clinical evidence in humans is still limited [101,102]. Cuproptosis-related transcriptomic signatures further stratify psoriasis into molecular subtypes with distinct immune infiltration patterns, supporting a contribution of copper-regulated pathways to disease heterogeneity and suggesting diagnostic or therapeutic potential, but these observations require functional validation [103].

In vitiligo, OS, LPO and weakened antioxidant defences are consistently reported in melanocytes, suggesting a redox-driven vulnerability [104]. Several studies propose ferroptosis as a plausible mechanism contributing to melanocyte loss: lesional skin shows reduced GPX4 and ferritin and increased transferrin receptor expression [105]. Recent transcriptomic analyses further identify SLC3A2 downregulation, the structural subunit of the system Xc^−^ Cys/Glu antiporter, as a driver of ferroptosis-related changes, including higher ROS, Fe^2+^, and LPO in vitiligo melanocytes [100,106]. Conversely, there is currently no evidence specifically addressing cuproptosis in vitiligo, so firm conclusions cannot yet be drawn.

In melanoma, ferroptosis and cuproptosis are both implicated in tumor biology. Ferroptosis-related pathways contribute to melanoma cell vulnerability to iron-dependent OS and to responses to targeted and immunotherapies. IFN-γ released by activated CD8^+^ T cells suppresses SLC7A11, the transport-active subunit of the system Xc^−^ Cys/Glu antiporter, and increases LPO, making melanoma cells more susceptible to ferroptotic killing during immune responses [107]. Other ferroptosis regulators—such as GPX4, ACSL4 and SLC7A11—also influence melanoma growth, metabolic adaptation and treatment resistance [108,109]. Conversely, cuproptosis is supported mainly by transcriptomic evidence. Multiple analyses show that cuproptosis-related genes and lncRNA signatures (including FDX1, LIPT1, and SLC31A1) define melanoma subgroups with distinct prognosis, immune infiltration patterns, and therapeutic sensitivity, in both cutaneous and uveal melanoma [110,111,112].

Across the cutaneous disorders discussed so far, a coherent pattern emerges in which ferroptosis and cuproptosis represent distinct yet partially convergent manifestations of cutaneous redox failure. In AD and psoriasis, parallel disturbances in LPO, iron handling, copper transport, and mitochondrial integrity indicate that keratinocytes may share upstream redox vulnerabilities that predispose them to both iron-dependent and copper-dependent cell-death pathways—with ferroptosis supported by robust functional evidence and cuproptosis suggested primarily by transcriptomic signatures [113]. In vitiligo, the absence of direct evidence for cuproptosis defines a key frontier for investigation, particularly given the disorder’s pronounced redox imbalance, metal dysregulation, and mitochondrial stress, which overlap mechanistically with upstream triggers of both ferroptosis and cuproptosis in other tissues [105]. In melanoma, both pathways are suggested to influence tumor biology through their effects on lipid oxidation, copper homeostasis, and Fe-S-cluster stability, contributing to metabolic adaptation, immune evasion, and therapeutic responsiveness [114]. Collectively, these observations position skin diseases as valuable systems for examining how iron- and copper-dependent death pathways may intersect within a broader redox-network framework, with implications for biomarker discovery and targeted therapeutic development.

### 3.3. Cancer and Chronic Inflammation

OS shapes tumor behavior by increasing cellular susceptibility to ferroptosis inducers and accelerating the onset of this iron-dependent death program under pathological conditions such as cancer [70]. Elevated ROS enhances the vulnerability of mesenchymal or dedifferentiated cancer cells—typically resistant to apoptosis and standard therapies—to ferroptotic death, supporting the view that ferroptosis represents an innate tumor-suppressive mechanism exploitable in refractory tumors [115,116,117,118].

Although tumor cells function under persistent redox imbalance with tightly regulated thiol–iron equilibrium, ferroptosis rarely occurs spontaneously during tumorigenesis, indicating that the process can eliminate transformed cells only under specific microenvironmental or metabolic constraints [119]. The ferroptotic cascade relies on Fe^2+^ accumulation, free-radical generation, disruption of antioxidant defenses such as GPX4, and extensive LPO, with regulators including ACSL4, SLC7A11, FTH1, and FSP1 shaping cancer sensitivity to this death pathway [115,120]. Depending on context, ferroptosis can either restrain tumor expansion or impair anticancer immunity, generating stage-dependent and microenvironment-dependent effects [115]. Reconciling this apparent paradox requires considering ferroptosis as a context-dependent process whose immunological consequences are dictated by timing, cellular composition, and the metabolic state of the TME [116]. Ferroptotic death of tumor cells can generally be advantageous for antitumor immunity, as oxidized phospholipids and DAMPs may enhance dendritic cell maturation, antigen presentation, and CD8^+^ T-cell priming [121]. However, under nutrient deprivation, hypoxia, and cys scarcity—features typical of the TME—effector lymphocytes themselves become highly susceptible to ferroptosis, leading to functional exhaustion and weakened cytotoxic surveillance. As a result, early ferroptosis in tumor cells supports immune activation, whereas late or sustained ferroptosis in infiltrating T cells, NK cells, or dendritic cells impairs antitumor immunity [116,122].

Beyond its immunological effects, ferroptosis interacts closely with other metal-driven death pathways within the TME, particularly cuproptosis.

In fact, both ferroptosis and cuproptosis remodel the TME through release of damage-associated molecular patterns, influencing metastasis, prognosis and therapeutic responsiveness [123]. Epigenetic regulators, including DNA methylation machinery, histone modifiers such as KDM3B, BAP1, PRC1 and LSH, and RNA-modifying enzymes, modulate ferroptosis and cuproptosis by altering expression of key death-associated proteins; dysregulation of ferroptosis-related genes is widespread in cancer, and SLC7A11 overexpression suppresses ferroptosis while promoting tumor growth [120]. Cuproptosis is driven by copper binding to lipoylated tricarboxylic-acid-cycle enzymes, causing aggregation of these mitochondrial proteins, destabilization of iron–sulfur cluster proteins and proteotoxic stress, ultimately inducing regulated cell death via a mechanism mechanistically distinct from other death programs [66,124]. Copper ionophores such as elesclomol deliver Cu^2+^ into mitochondria, where reduction via FDX1 to Cu^+^ elevates ROS and triggers cuproptotic death, establishing a functional connection between copper homeostasis and OS [124]. Tumor susceptibility to copper-dependent death can be therapeutically exploited by pharmacologic modulation of intratumoral copper availability, and cuproptosis is increasingly recognized as an important factor in cancer progression and treatment response [124]. Copper regulates immune signaling, as induction of cuproptosis activates the cyclic GMP–AMP synthase–stimulator of interferon genes (cGAS–STING) pathway, increases the production of interleukin-2 (IL-2), tumor necrosis factor-α (TNF-α), interferon-γ (IFN-γ), and the chemokines CXCL10 and CXCL11, and enhances the efficacy of anti–programmed cell death protein 1 (PD-1) therapy by promoting the expansion of CD8^+^ T-cell populations in tumor-bearing hosts [124]. Copper also modulates immune-checkpoint biology: its addition increases programmed death ligand 1 (PD-L1) expression, whereas copper chelation decreases PD-L1 stability and promotes infiltration of CD8^+^ T cells and NK cells [124]. Cuproptosis-related gene and lncRNA signatures correlate with prognosis and the immune landscape across multiple cancers, highlighting the relevance of copper-dependent death in tumor evolution [120]. OS-responsive nanoplatforms designed to amplify ROS can potentiate cuproptosis-based combination therapies, and nanoparticles co-delivering elesclomol, copper, and PD-L1 blockade enhance cuproptosis and improve immunotherapeutic outcomes [124].

### 3.4. Neurodegenerative Disorders

OS represents a fundamental pathogenic mechanism in neurodegenerative diseases, where the brain’s elevated metabolic demands and limited antioxidant buffering capacity lead to a biochemical environment highly susceptible to redox imbalance [125]. In several neurological disorders, the accumulation of ROS exceeds detoxification pathways, driving extensive LPO that destabilizes neuronal membranes and promotes regulated cell death [125].

In central nervous system disorders, nerve cell ferroptosis is reported to be driven by iron overload, Fenton reaction–mediated ROS generation, LPO, mitochondrial atrophy and functional decline, ultimately impairing neuronal energy supply and contributing to cognitive dysfunction [126]. Increased levels of LPO products such as MDA and 4-HNE, together with GSH depletion, are associated with blood–brain barrier disruption, highlighting the relevance of redox imbalance and ferroptosis-related pathways in central neurological diseases [126,127,128]. Consistently, inhibitors of ferroptosis such as ferrostatins and liproxstatins have been shown to protect from ischemic injury in the brain and to ameliorate pathology in models of degenerative brain disorders, including Parkinson’s, Huntington’s and Alzheimer’s diseases, as well as in traumatic and hemorrhagic brain injury [125,129,130].

Copper-related OS and cuproptosis are specifically implicated in Alzheimer’s disease [131,132]. Free or loosely bound copper is reported to be increased in serum and brain of patients with cognitive impairment and Alzheimer’s disease, where it reduces antioxidant defenses, increases MDA and other LPO markers, and induces mitochondrial dysfunction in hippocampal tissue [133]. These studies indicate that excessive copper can induce oxidative damage, neuronal death, and astrocyte proliferation in the hippocampus, leading to impaired learning and memory [133]. In Alzheimer’s disease, pathological copper accumulation in cortical regions and within amyloid-β plaques further exacerbates OS by catalyzing Fenton-like reactions, depleting GSH, thus amplifying mitochondrial dysfunction [134]. Cuproptosis is described as a copper-dependent programmed cell death distinct from apoptosis, ferroptosis, and necroptosis, mediated by mitochondrial protein lipoylation and involving aggregation of lipoylated tricarboxylic-acid-cycle enzymes, loss of Fe-S cluster proteins, heat-shock-protein-70 induction, and acute proteotoxic stress [133]. In the context of Alzheimer’s disease, excessive copper is proposed to promote neurodegeneration by inducing cuproptosis together with OS, synaptic damage, amyloid-β plaque deposition and neuronal death, thereby linking copper dyshomeostasis to disease progression [133,134,135]. However, most data implicating cuproptosis in neurodegenerative disorders derive from experimental models, and direct demonstration of canonical cuproptosis in human tissues is still lacking [136].

### 3.5. Cardiovascular Diseases

A growing body of evidence demonstrates that dysregulated iron and copper homeostasis may contribute to the pathogenesis of major cardiovascular diseases (CVDs), in part through ferroptosis- and cuproptosis-related mechanisms. Ferroptosis has been implicated in hypertension, atherosclerosis, pulmonary hypertension, myocardial ischemia–reperfusion injury, cardiomyopathy, and heart failure [136].

In these conditions, excess intracellular iron enlarges the labile Fe^2+^ pool, facilitating Fenton-type ROS generation and the peroxidation of PUFA-containing phospholipids in cardiomyocyte membranes. The resulting accumulation of lipid hydroperoxides disrupts membrane integrity and mitochondrial function, while GPX4 loss or insufficiency further accelerates redox collapse [137]. In support of this, ferroptosis inhibition through iron chelation, radical-trapping antioxidants, or GPX4-supporting pathways has been shown to reduce cardiomyocyte injury and attenuate pathological cardiac remodeling, indicating that iron-driven LPO is a well-supported mechanistic contributor to cardiovascular pathology [136].

Copper-dependent cell death has likewise emerged as a pathogenic process in vascular and metabolic cardiac disease [138]. Excess intracellular copper binds to lipoylated mitochondrial TCA-cycle enzymes, leading to proteotoxic stress, Fe-S cluster destabilization, and mitochondrial failure—a mechanism established in cuproptosis and increasingly supported by experimental models of atherosclerosis, ischemia–reperfusion injury, stroke, and heart failure, rather than fully demonstrated in human cardiovascular tissues [139].

Copper overload enhances OS, triggers endothelial dysfunction, and impairs the ubiquitin–proteasome system, thereby amplifying vascular damage and inflammation [139,140].

Copper deficiency may also compromise vascular elasticity and increase platelet aggregation, thus predisposing to ischemic cardiovascular events [139]. These copper-dependent effects on mitochondrial metabolism and vascular homeostasis are supported by experimental observations.

Emerging cardiovascular evidence suggests that ferroptosis and cuproptosis, while initiated through distinct iron- and copper-dependent triggers, may converge mechanistically at multiple metabolic and mitochondrial nodes. Recent analyses suggest that copper dysregulation may modulate ferroptotic susceptibility by altering iron handling: copper acts as a cofactor for iron-metabolizing enzymes, and both copper deficiency and copper overload reshape transferrin-mediated iron uptake, ferroportin activity, and Fe-S-cluster integrity, thereby influencing the size and reactivity of the labile iron pool [67]. Conversely, Fe-S-cluster loss induced by copper overload promotes mitochondrial Fe^2+^ accumulation and secondary amplification of ferroptotic ROS. Mitochondrial TCA cycle seems to be a shared regulatory hub: lipoylation-dependent cuproptosis and glutamine-driven mitochondrial potentiation of ferroptosis both require intact oxidative metabolism; inhibition of mitochondrial respiration suppresses cuproptosis and attenuates ferroptotic ROS amplification, suggesting a common metabolic vulnerability [136]. Additionally, intracellular GSH depletion acts as a biochemical intersection, since ferroptosis inducers impair GPX4 function while simultaneously reducing copper-chelation capacity, thereby enhancing cuproptotic susceptibility [141]. Despite these emerging mechanistic links, the in vivo relevance and quantitative contribution of ferroptosis–cuproptosis cross-talk in human cardiovascular disease remain incompletely defined. Most integrative insights derive from transcriptomic, computational, or in vitro studies, and dedicated in vivo models are required to clarify whether these interactions represent accessory phenomena or core drivers of cardiometabolic injury [69].

### 3.6. Infectious Diseases

OS is both a host defense strategy and a driver of tissue injury during infections. Pathogens and immune cells compete for control of metal redox fluxes—particularly iron and copper—that determine whether oxidative signaling promotes microbial clearance or collateral damage. Ferroptosis is now recognized as a double-edged mechanism in infectious disease. For example, during *Mycobacterium tuberculosis* (M.tb) infection, ferroptosis limits intracellular bacterial growth by restricting iron availability and limiting bacterial replication. However, excessive ferroptosis amplifies ROS production, disrupts membrane integrity, and releases DAMPs that fuel necrosis and bacterial dissemination [142]. While ferroptosis has clear functional effects in infection models, evidence for canonical cuproptosis in infectious settings remains preliminary. In infected macrophages, copper accumulation acts as an antimicrobial mechanism, since phagosomal copper can damage *M. tuberculosis* by disrupting iron–sulfur cluster proteins and promoting oxidative injury. However, *M. tuberculosis* counters copper toxicity through efflux pumps and metallochaperones that maintain intracellular redox balance and support virulence. Although excessive host copper could theoretically overwhelm these defences and enhance bacterial control, direct evidence for canonical cuproptosis in TB or for copper-driven tissue injury remains limited and largely exploratory [142].

In sepsis, copper dysregulation and mitochondrial stress converge to trigger both ferroptotic and cuproptotic cascades. Specifically, ferroptosis contributes to organ injury by intensifying LPO, disrupting iron homeostasis, and amplifying inflammatory signalling pathways. Increased ferroptotic activity correlates with worse clinical outcomes, while pharmacologic inhibition mitigates lung, renal and myocardial damage in experimental models, supporting a pathogenic role in sepsis-associated organ dysfunction [143,144,145]. Cuproptosis has also emerged as a relevant process: sepsis is characterized by disturbed copper handling, mitochondrial injury, and enhanced protein lipoylation, all of which favour copper-dependent cytotoxicity. Elevated circulating or intracellular copper levels associate with increased oxidative burden, impaired mitochondrial metabolism, and adverse prognosis in septic patients, while experimental modulation of copper availability can attenuate tissue damage [146,147]. Collectively, dysregulated iron- and copper-driven cell-death pathways appear to converge in sepsis, reinforcing OS and multi-organ dysfunction, and represent emerging targets for therapeutic intervention.

In infectious disease settings, ferroptosis and cuproptosis may contribute to a coordinated metal-based host defence strategy while also posing risks for collateral tissue injury [45,63]. Iron restriction and ferroptotic stress may suppress pathogen expansion, whereas copper accumulation within phagosomes disrupts microbial Fe-S-cluster proteins and mitochondrial-like metabolism [148]. In sepsis, combined disturbances in iron and copper handling may amplify mitochondrial dysfunction, ROS production, and systemic inflammation, highlighting the possibility that ferroptotic and cuproptotic mechanisms operate in parallel within the same tissues [141,146,147].

### 3.7. Systemic Redox Crosstalk

Growing evidence indicates that ferroptosis and cuproptosis—although mechanistically distinct—share upstream metabolic and mitochondrial vulnerabilities that may permit partial biochemical convergence under oxidative or metabolic stress [67]. The most established interaction node involves mitochondrial metabolism: both pathways require intact TCA-cycle flux and oxidative phosphorylation. In cuproptosis, mitochondrial Cu^+^ binds aberrantly to lipoylated TCA-cycle enzymes, driving proteotoxic aggregation and destabilizing Fe-S cluster proteins; the resulting release of Fe^2+^ can amplify Fenton-type LPO and lower the threshold for ferroptotic initiation [65]. Conversely, ferroptosis-associated mitochondrial ROS may intensify copper-mediated proteotoxic stress, suggesting a bidirectional amplification loop; however, this latter relationship is supported primarily by preclinical models and remains incompletely validated in vivo [139].

A second, functionally plausible but still emerging point of intersection involves GSH homeostasis. GSH depletion is a well-established requirement for ferroptosis due to impairment of GPX4-dependent detoxification of phospholipid hydroperoxides. Because GSH also acts as a major intracellular Cu^+^ buffer, reduced thiol availability may increase the pool of redox-active copper capable of engaging the lipoylated proteome, thereby enhancing susceptibility to cuproptosis [136]. Although this mechanism is supported by biochemical studies, its quantitative contribution in vivo remains to be elucidated.

A third convergence point involves Fe/Cu homeostatic interplay. Copper overload destabilizes Fe-S clusters, increases mitochondrial labile Fe^2+^, and impairs iron–sulfur protein maturation—processes with strong experimental support—thereby enhancing LPO and ferroptotic sensitivity. In parallel, iron overload increases mitochondrial ROS and perturbs copper redox cycling, potentially intensifying cuproptotic triggers [69]. While these metal-interaction pathways are mechanistically coherent and individually well documented, their coordinated operation within human tissues remains an active area of investigation.

Finally, despite diverging execution mechanisms—LPO in ferroptosis versus proteotoxic aggregation in cuproptosis—both pathways converge downstream on mitochondrial dysfunction, ROS escalation, and DAMP release, with shared implications for inflammatory signalling [67].

Collectively, these observations support a conceptual framework in which ferroptosis and cuproptosis may arise as parallel yet interconnected manifestations of metal-driven redox-network destabilization. Nevertheless, most integrative models of Fe-S cluster instability, ROS amplification loops, and GSH-dependent crosstalk are derived from preclinical, in vitro, or computational analyses. Direct evidence for coordinated activation of these pathways in vivo remains limited, and the proposed interactions should therefore be interpreted as evolving mechanistic hypotheses (Table 1).

## 4. Clinical and Therapeutic Possible Applications

Markers of LPO and OS are increasingly discussed as potential clinical readouts across different diseases. OS–induced ferroptosis is linked to a wide spectrum of conditions, including cardiological, neurodegenerative, cutaneous and autoimmune diseases, infections and inflammation [70].

Ferroptosis has already been clearly linked to liver disease, cardiovascular diseases (including atherosclerosis, drug-induced heart failure, myocardial ischaemia–reperfusion injury, sepsis-induced cardiomyopathy, arrhythmia and diabetic cardiomyopathy), Alzheimer’s disease, cerebral ischemia–reperfusion injury and multiple sclerosis [137]. Beyond playing a role in these conditions, ferroptosis inhibition is being studied as a possible strategy that can protect against pathologies. For example, in cardiomyopathy, in experimental models, some evidence supports the concept that anti-ferroptotic interventions may become organ-protective therapies in cardiac settings [137].

Ferroptosis and cuproptosis have emerged with clear translational relevance in cancer, with implications for therapy response, resistance, and drug development. Tumor cells often display heightened iron and copper dependency, altered mitochondrial metabolism, and disrupted redox homeostasis, creating a metabolic environment that can be exploited therapeutically. Ferroptosis contributes to chemosensitivity, radiosensitivity, and immunotherapy responsiveness: induction of iron-dependent LPO can overcome resistance to agents such as oxaliplatin, cisplatin, anthracyclines, and temozolomide, while ferroptosis inducers (e.g., erastin, RSL3) enhance the efficacy of chemotherapy and radiotherapy in preclinical models. Conversely, ferroptosis resistance mechanisms—iron sequestration, GPX4 stabilization, SLC7A11 upregulation—are increasingly recognized as drivers of treatment failure. Cuproptosis, driven by copper accumulation and lipoylated TCA-cycle protein aggregation, also shows therapeutic promise. Copper ionophores such as elesclomol and disulfiram can selectively kill cancer cells, including chemoresistant subsets, and synergize with ferroptosis inducers through shared mitochondrial and GSH-dependent pathways. Cuproptosis-related genes (FDX1, DLAT, LIAS, SLC31A1) have prognostic value and correlate with immunotherapy sensitivity and tumor immune infiltration. Nanoparticle-based co-delivery systems that simultaneously activate ferroptosis and cuproptosis demonstrate enhanced antitumor efficacy with reduced off-target toxicity. Altogether, coordinated targeting of ferroptosis and cuproptosis represents a promising avenue for overcoming drug resistance and advancing precision oncology, though translation to clinical practice requires optimization of specificity and safety [149,150,151].

Despite their therapeutic promise, ferroptosis- and cuproptosis-targeted strategies face several limitations. Systemic copper modulation remains problematic: ionophores such as elesclomol and disulfiram can induce mitochondrial stress, cardiotoxicity, and neurotoxicity [152,153]. Copper chelators also pose safety concerns—trientine may cause hypotension and neurological worsening, D-penicillamine affects vascular integrity, and metal redistribution can contribute to neuro- and hepatotoxicity [139,154]. Even tetrathiomolybdate may disrupt systemic copper homeostasis with chronic use [155].

A second limitation is the lack of selective biomarkers. Classical ferroptosis markers (MDA, 4-HNE, ACSL4 upregulation, GPX4 loss, iron accumulation) lack specificity, while cuproptosis indicators—lipoylated DLAT aggregation, Fe-S cluster depletion, HSP70 induction—remain confined to research settings [152,156]. A final barrier concerns long-term safety. Several ferroptosis-inducing agents show nephrotoxicity, neurotoxicity, and poor biocompatibility, and most cuproptosis-modulating approaches remain preclinical, with uncertain cardiovascular or systemic safety in copper-dysregulated states [67,152,157]. Collectively, these issues highlight the need for safer metal-targeting strategies, improved delivery systems, and clinically applicable biomarkers.

At the molecular level, epigenetic and transcriptomic signatures involving ferroptosis-related genes—such as a ferroptosis-associated DNA methylation signature predicting overall survival in head and neck squamous cell carcinoma, and a ferroptosis-related DNA methylation signature reported to predict prognosis and guide treatment in cutaneous melanoma—demonstrate that ferroptosis-linked gene sets can function as prognostic and treatment-oriented biomarkers in clinical cancer cohorts [158,159,160].

For cuproptosis, a recent study analyzed cuproptosis-related genes, described their expression patterns across multiple tumor types, and their impact on patient outcomes, emphasizing the translational potential of copper chelators, copper ionophores, and copper-based nanomaterials as therapeutic tools, together with the remodeling of the tumor microenvironment and enhancement of immune cell infiltration [124,134]. In parallel, it has been highlighted that copper homeostasis is crucial for mitochondrial function and antioxidant defense, discusses copper dysregulation in conditions such as Wilson’s disease and brain disease, and frames cuproptosis as a disease-relevant, copper-dependent regulated cell death pathway whose biomarkers and modulators—chelators as “anti-cuproptosis” agents and ionophores as inducers—have clear potential for future diagnostic and therapeutic use in oncology and in disorders characterized by copper overload or mitochondrial dysfunction [66,124,134].

Moreover, artificial intelligence (AI) is revolutionizing how OS–related processes, particularly LPO, ferroptosis, and cuproptosis, are studied, predicted, and clinically applied. Machine learning (ML) algorithms and neural networks allow for the integration of multi-omics datasets (lipidomics, metallomics, transcriptomics) to uncover patterns that are invisible to traditional statistical analysis. This computational approach is demonstrating significant clinical utility in diagnosing and predicting outcomes across diseases where redox imbalance is a driving force [161,162].

In the context of LPO, artificial neural network (ANN) models have been successfully used to detect subtle oxidative signatures in complex biological matrices. On this topic, Peña-Bautista et al. showed that ANN-based classification of LPO compounds in plasma and urine outperformed classical linear models (ROC-AUC > 0.88), offering high diagnostic accuracy for early Alzheimer’s disease [163].

Furthermore, the role of AI in cuproptosis research is expanding rapidly. Luo et al. applied multiple ML algorithms to transcriptomic data from COVID-19 patients, identifying two cuproptosis-related molecular subtypes with distinct immune microenvironment profiles. Their “cuproptosis-related risk score” (CRRS) achieved high predictive accuracy for disease severity and prognosis [164]. Similarly, Cai et al. developed an ML–derived nomogram using eight cuproptosis-related genes (e.g., KLF5, GNL3, ALAS1) to predict hepatic ischemia–reperfusion injury, achieving an AUC of 0.97 [165]. Both studies exemplify how AI can integrate gene expression, immune infiltration, and metal homeostasis data into clinically interpretable predictive tools.

In summary, AI may enable the transition from descriptive redox biology to predictive, systems-level modeling of oxidative cell death. By capturing nonlinear interactions among lipids, metals, and metabolism, AI platforms not only refine diagnostic precision but may also guide therapeutic innovation.

## 5. Conclusions and Future Perspectives

Redox biology has entered a new era in which OS is no longer viewed as a nonspecific marker of damage but as an organized network that integrates metabolism, immunity, and metal homeostasis. Ferroptosis and cuproptosis exemplify how dysregulated iron and copper fluxes convert adaptive redox signaling into regulated cell death, providing a mechanistic bridge between metabolic dysfunction, inflammation, and tissue degeneration. The convergence of these pathways across endocrine, dermatologic, cardiovascular, and neurologic systems underscores the systemic nature of redox miscommunication and its central role in human disease.

Future research should move beyond descriptive correlations toward mechanistic mapping of the redox network at the organismal level. Integrating redox lipidomics, metallomics, and proteomics with machine learning will enable the construction of dynamic “redox phenotypes” that predict disease trajectory and therapeutic response. Clinically, combining ferroptosis and cuproptosis modulation with immunometabolic reprogramming represents a promising frontier for precision medicine. Ultimately, redefining OS as a quantifiable and therapeutically tractable parameter may transform it from a retrospective hallmark of injury into a proactive biomarker of health and resilience.

## Figures and Tables

**Table 1:** The principal biochemical nodes at which ferroptosis and cuproptosis converge are summarized.

Shared Mechanism	Contribution of Ferroptosis	Contribution of Cuproptosis	Link Between Ferroptosis and Cuproptosis
**Mitochondrial Fe-S cluster instability**	Oxidative degradation of Fe-S clusters increases mitochondrial Fe^2+^ and ROS	Cu^+^ binding destabilises Fe-S clusters and releases Fe^2+^	Cuproptosis-induced Fe^2+^ release lowers the ferroptosis threshold; ferroptotic ROS exacerbate cuproptotic proteotoxicity [65,125]
**GSH depletion/thiol-redox collapse**	GSH loss → GPX4 inactivation → LPO	GSH loss reduces Cu^+^ buffering → more free copper to engage lipoylated proteins	Competition for GSH links the pathways: ferroptosis inducers sensitize cells to cuproptosis [30,31]
**Fe/Cu homeostatic interplay**	Iron overload drives Fenton ROS and enhances LPO	Copper overload impairs Fe-S cluster assembly, increasing mitochondrial Fe^2+^	Copper-driven Fe^2+^ release amplifies ferroptosis, while iron dysregulation perturbs copper redox cycling [67,69]
**TCA-cycle dependency and mitochondrial metabolism**	Mitochondrial ROS and glutaminolysis potentiate LPO	Lipoylation-dependent TCA enzyme aggregation drives cuproptosis	Both require intact oxidative metabolism; inhibition of mitochondrial function protects from both [65]
**Downstream oxidative/inflammatory signalling**	DAMP release via oxidized lipids activates innate immunity	Proteotoxic stress activates mitochondrial danger pathways	Shared inflammatory amplification from distinct upstream triggers [32,70]

## Data Availability

No new data were created or analyzed in this study. Data sharing is not applicable to this article.
